# Facilitators and barriers to physicians’ entrepreneurial ventures in major Japanese cities: A qualitative study

**DOI:** 10.1371/journal.pone.0258957

**Published:** 2021-10-27

**Authors:** Daichi Yashiro, Nobutoshi Nawa, Eriko Okada, Hiroaki Kato, Sarara Yonemori-Matsumoto, Ayako Kashimada, Yasuhiro Itsui, Yujiro Tanaka

**Affiliations:** 1 Department of Medical Education Research and Development, Tokyo Medical and Dental University, Tokyo, Japan; 2 Professional Development Center, Tokyo Medical and Dental University, Tokyo, Japan; University of Botswana Faculty of Medicine, BOTSWANA

## Abstract

**Background:**

The Japanese healthcare system currently faces numerous challenges, including a super-aging society and an excessive burden on medical workers; therefore, the need for innovative solutions from healthcare ventures to tackle these issues has increased. Meanwhile, as physicians play important roles in healthcare ventures, the need for Japanese physician entrepreneurs is more important than ever. Given the lack of research examining barriers to physicians starting ventures and what skills, knowledge, and surrounding environments act as facilitators, this study aimed to identify the facilitators and barriers faced by physicians to start ventures.

**Methods:**

Between September and November 2019 and in May 2021, qualitative interviews were conducted with 33 participants, which included eight physician entrepreneurs; two administrative officers at the Ministry of Health, Labour and Welfare and the Ministry of Economy, Trade and Industry; three faculty members at Tokyo Medical and Dental University (in-depth interviews); and 20 medical students (focus group discussions). The interviews were deductively coded based on the social ecological model. The inductive approach was applied to coding any knowledge necessary to start a business. We conducted member checking with three physician entrepreneurs and seven medical students to improve our results’ credibility.

**Results:**

The factors influencing a physician’s decision to launch a new business include their willingness to contribute to society, the unique environment in which an individual is placed while in medical school and afterward, negative aspects of the lack of diversity in physicians’ careers, the financial stability provided by a medical license, and self-efficacy.

**Conclusions:**

Our study revealed facilitators and barriers to physicians’ entrepreneurial ventures. Knowledge about these factors might be useful in supporting physicians to launch or become involved in healthcare ventures.

## Introduction

In Japan, the demand for physician entrepreneurs is more important than ever. As the Japanese healthcare system faces numerous challenges, including a super-aging society and an excessive burden on medical workers [1], the need for innovative solutions from healthcare ventures to tackle these issues is increasing [2,3]. Physicians can play an important role in healthcare ventures, because they can provide clinical perspectives essential to needs assessment, product design, prototyping, and evaluation of a product’s usability [4]. Furthermore, physicians can devise clinical trials to evaluate the product’s efficacy by themselves or by leveraging their connections with other healthcare providers. In response to this heightened need, the Ministry of Health, Labour, and Welfare and the Ministry of Economy, Trade, and Industry in Japan recently launched programs to support such ventures [5,6].

To adequately support physicians to launch or become involved in healthcare ventures, understanding the factors that facilitate or hinder their entrepreneurial behaviors is crucial. However, studies on facilitators and barriers to physicians’ entrepreneurial ventures are currently limited [7]. One study conducted in the US examined the characteristics of physician entrepreneurship in Massachusetts and reported that male physicians (compared with female physicians) and attendees of the top 10 medical schools in the United States (ranked according to National Institute of Health funding) are more likely to start a new company [7]. Previous reports on barriers to nurses starting healthcare-related ventures identified barriers such as the lack of business knowledge and skills, concerns regarding legal issues, lack of a mindset for starting a business, and perceptions that "nursing is about care, whereas business is about making money” [8,9]. Other studies focusing on general entrepreneurs, rather than physician entrepreneurs have reported that factors such as a proactive personality [10,11], the existence of a role model [12], and experiences and knowledge of launching new ventures [13–15] were associated with entrepreneurs’ intention.

These studies on individual factors associated with entrepreneurs’ intention may be important to understand how to promote entrepreneurial behaviors among physicians. However, on the basis of the social ecological model [16–18], individual behaviors are influenced by factors at the individual level as well as interpersonal, organizational, community, and societal levels. Although, to our knowledge, there have been no studies on the contextual factors of physician entrepreneurs, numerous studies have been conducted focusing on general entrepreneurs in healthcare ventures [19–21]. Contextual factors that influence entrepreneurship include conservative and reserved norms [21]; hesitancy of healthcare providers to adopt new technologies [21]; perceived competition; organizational support for entrepreneurs; and an entrepreneurial environment with high levels of autonomy, time availability, and collaboration [20]. In fact, one study reported lower acceptance of valuable innovations from those outside the healthcare system [21]. Although these results are interesting, it is possible that facilitators and barriers for physician entrepreneurs, who are insiders of the healthcare system, may differ from those of entrepreneurs in general. Thus, studies assessing factors that promote or inhibit entrepreneurs’ intention operating at multiple levels is crucial. The objective of this study was to clarify factors that promote or inhibit physicians from starting ventures.

## Materials and methods

### Sample

We conducted a qualitative survey in 33 participants, which included eight physician entrepreneurs; two administrative officers at the Ministry of Health, Labour, and Welfare and the Ministry of Economy, Trade, and Industry; three faculty members at Tokyo Medical and Dental University; and 20 medical students at Tokyo Medical and Dental University.

We used purposeful sampling to conduct a qualitative survey with “information-rich” participants [22]. More specifically, we used snowball sampling and first approached the initial participants through our personal connections. Then, we approached the information-rich participants based on their introductions.

Three physician entrepreneurs were contacted through personal connections of the authors as initial physician entrepreneur participants. Subsequently, the remaining five physician entrepreneurs were recruited via the initial participants and contacted through snowball sampling. All the physician entrepreneurs were recruited via email except for two who were recruited in person. A total of 23 medical students at Tokyo Medical and Dental University considered by the authors to be particularly knowledgeable about the topic were recruited in person through authors’ academic connections (the authors are faculty members of the university) and personal connections (the first author is a medical student) and 17 agreed to participate in the focus group discussions (FGDs). In addition, the first author (a medical student) used SNS to approach 110 medical students at Tokyo Medical and Dental University and invited those who were interested in this study to participate in FGDs; an additional three medical students agreed to participate. In this study, physician entrepreneurs and medical students were considered primary study participants, whereas administrative officers and faculty members were considered key informants. Key informant participants (three faculty members and two administrative officers) were contacted by email through personal connections of the authors. Owing to the limited sample size of key informants, the results are focused on the primary study participants, and the findings on the key informants are provided in a separate section in the results. The perspectives of the key informant participants were checked to determine whether important findings can be added to the results; these are then listed in a separate section. All participants provided written informed consent before participating. This study was approved by the Research Ethics Committee of Tokyo Medical and Dental University (approval M2019-118).

### Data collection

After obtaining written informed consent, we used the interview guide to conduct in-depth interviews (IDIs) with each entrepreneur, administrative officer, and teacher, each lasting approximately 60 minutes. Except for one online interview and a face-to-face interview in the cafeteria, IDIs were conducted mainly in university conference rooms or in the offices of physician entrepreneurs. We also conducted four focus group discussions with medical students, two online and two face-to-face in a conference room at Tokyo Medical and Dental University. We used an audio recorder to record interviews and focus group discussions. Initial interviews and FGDs were conducted between September and November 2019 in Japanese. Additional FGDs were held in May 2021, the results of which confirmed results of earlier IDIs and FGDs. YD, NN, and YI conducted the qualitative interviews. NN received formal training in qualitative interviewing in a graduate program and led the quality assurance of interviews, which included training interviewers. Our interview guide for entrepreneurs comprised questions on their age; career; background of ventures; what they considered were obstacles to launching ventures; the knowledge, skills, and support they thought were facilitators and were effective in reducing obstacles; and appropriate content and duration of classes to support physicians or medical students starting businesses. Our interview guide for medical students included questions on what they considered were obstacles to launching ventures; the knowledge, skills, and support they thought were facilitators and were effective in reducing obstacles; and the grade in which they want to receive entrepreneurship education (e.g., during liberal arts, before clinical practice, and after clinical practice). The interview guide for administrative officers included questions requesting their advice on support programs that Japanese universities could offer (e.g., graduate programs for physicians and MBA programs), features of the ministry’s current programs [5,6], strength of the programs, similar programs in other countries, and programs under development. Faculty members were interviewed about the types of classes that should be provided to motivate medical students to become more entrepreneurial and how the current curriculum could be changed. Please see the S1 File for the interview guide.

Reflexivity was considered throughout the data collection and analyses [23]. The medical students who participated in the survey knew that most authors were faculty members and physicians (only the first author was a medical student). To prevent this power relationship from biasing the responses, the first author (medical student) took the lead in interviewing the medical students. Further, during the interview, care was taken to explain that the interview data would be anonymized and reported such that individuals would not be identified, and thus, they could freely express their opinions. Further, because the authors, medical students and physicians, interviewed medical students and physician entrepreneurs are in the same medical field, it was possible that some opinions were omitted and not mentioned because the interviewees thought that we would share the same knowledge. Therefore, when the intention of a statement was unclear, we tried to check the meaning.

### Data analyses

All interview data in Japanese were transcribed verbatim. Next, thematic analysis was performed [24]. Following the methods of previous studies [25–28], DY and NN first coded one part of the interview data to create an initial codebook, and DY, NN, EO, and YT then discussed, revised, and finalized the codebook. DY led the coding process based on the codebook, and DY and NN met every 2–3 days to discuss problems that arose when applying the code. The final code list was established during discussions in a research meeting (e.g., which codes should be integrated into the list of final codes). On the basis of the social ecological model, codes were divided into individual, relationship, community, and social levels [16–18]. When the codes and quotes were reported, native Japanese speakers who were also fluent in English (NN and DY) translated the quotes and codes into English, and native English speakers checked the accuracy of the English wording and made changes when necessary without altering the original meaning. NN and DY checked the changes, and all authors gave final approval of the content.

Reflexivity was also considered during coding [23], and we tried not to make excessive interpretations of what was said. To ensure that the key themes were not missed and check the credibility of our interpretation, we conducted member checking with three physician entrepreneurs with whom we conducted IDIs and seven medical students with whom we conducted FGDs by showing them the final results of our analysis [29]. All of them indicated that the interpretation resonated with their experiences.

The interview data were organized using the basic folder function of Microsoft Windows, the codebook was created in Microsoft Excel, and the coding was performed in Microsoft Word.

## Results

Table 1 presents the participants’ characteristics. All entrepreneurs were male, with a mean age of 35.7 years and 5 years of clinical work experience before launching their business. Of the 20 medical students who participated in the FGD, 12 (60%) were male. Both administrative officers were male; two of the three faculty members were male and one was female.

**Table 1:** Characteristics of physician entrepreneurs.

Variable		Average or number	SD or %
**Entrepreneur**			
**Sex**	Male	8	100
	Female	0	0
**Mean age, years**		35.7	4.8
**Mean years of clinical work (including residency)**		5.1	2.6
**Medical students**			
**Sex**	Male	12	60
	Female	8	40
**Grade**	6th	1	5
	5th	2	10
	4th	9	45
	3rd	3	15
	2nd	5	25
**Administrative officers**			
**Sex**	Male	2	100
	Female	0	0
**Faculty members**			
**Sex**	Male	2	66.7
	Female	1	33.3

### Vignettes

**Fig 1:**
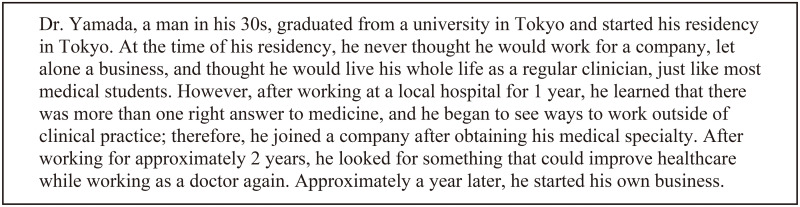
Case 1: Dr. Yamada’s story. (Pseudonym was used to protect participant privacy).

**Fig 2:**
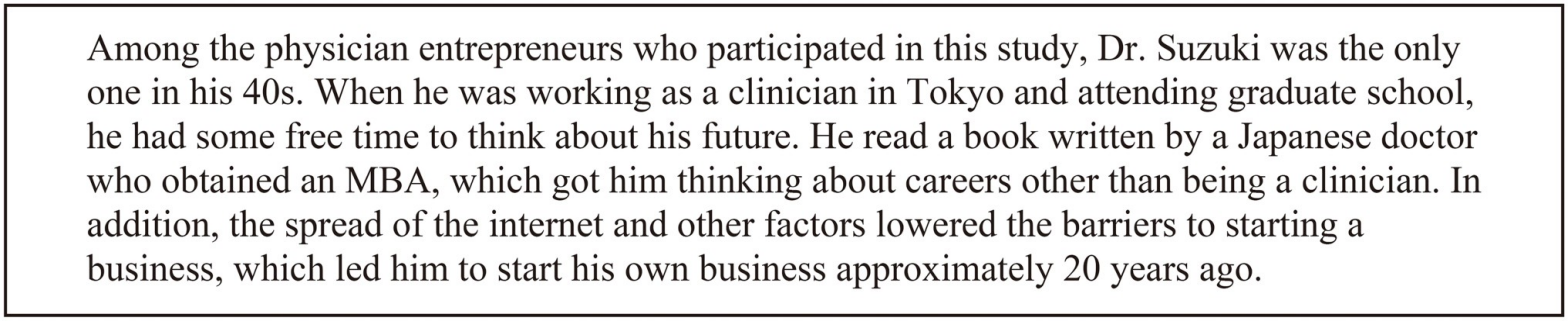
Case 2: Dr. Suzuki’s story. (Pseudonym was used to protect participant privacy).

The details of some of the physician entrepreneurs are presented as vignettes in Figs 1 and 2.

### Facilitators

Based on the social ecological model, we classified the 11 codes into four levels (i.e., individual, relationship, community, and societal) [16–18].

#### Individual

*Will to contribute more to society*. Most physician entrepreneurs in this study mentioned their will to contribute more to society. In contrast to the clinical work that is a traditional physician’s career, physician entrepreneurs chose business as a way to contribute to society. For example, one of the physician entrepreneurs noted, “When I was a medical student, I established a publishing firm. After becoming a physician, I believed that my work as a physician may have lower impact on the society than that of a publisher” [PE7].

*Prior experiences of challenge and success*. Similarly, physician entrepreneurs repeatedly mentioned their experiences of challenge and success. They thought that overcoming their past challenges could be a facilitator in launching a new business, as they gained confidence in their own abilities. One physician entrepreneur stated, “Most of my friends who are alumni of the Stanford Graduate School of Business had backpacked in the past. I believe that those who attempted something adventurous are more likely to show interest in starting a new business” [PE7].

*Self-efficacy*. Self-efficacy is the confidence in breaking through difficulties and succeeding in challenging tasks [30]. In the context of entrepreneurship among physicians, physician entrepreneurs noted that if physicians believe in their ability to launch a new business, they can more easily face the challenges of starting a business. Some entrepreneurs referred to this topic, which relates to the common misunderstanding that starting a business is something only gifted people can do. The following statement expresses this theme well: “It may be important to inform medical doctors and students that [if they try to launch a new business] they can actually succeed and handle social problems concerning medical care” [PE2].

*Identification of key medical problems*. All physician entrepreneurs interviewed in this study identified key medical problems encountered during their clinical work that they really wanted to solve, and they chose business as the best way to solve them. An entrepreneur said, “I believe that the physicians’ practice for patients with symptoms suspected of influenza should be improved. Owing to the current limitation in diagnostic tools, it is sometimes difficult to diagnose influenza in its early stages” [PE2].

*Having a wide range of interests*. Some entrepreneurs regarded having a wide range of interest to be an important individual characteristic. They mentioned that the more physicians are interested in a variety of things, especially topics unrelated to medicine, the more likely they are to launch a new business. One respondent noted, “What made me believe that I might be better suited to become an entrepreneur than other doctors was my interest in a variety of topics. When you’re interested in a variety of things, you’re more likely to observe problems and solutions from unexpected perspectives” [PE5].

*Recognition that little knowledge about business is needed*. Physician entrepreneurs stated that physicians often hesitate to launch a business because they believe that it requires substantial business knowledge that they do not possess. However, some physician entrepreneurs indicated that it was not too late to learn on the job if needed. One participant insisted that “It might have been easier if I had an MBA degree, but I did not care at all [about the lack of knowledge in the early stages] because I believed that I could learn what I needed to know whenever I needed it” [PE1].

#### Relationship

*Connection with other professions*. All physician entrepreneurs underscored the importance of connection with other professions. This connection can provide physicians or medical students with various resources so that they can choose other ways to achieve their goals apart from their physician’s career. One respondent stated, “In general, a university student will have the option of establishing a business while interacting with people from various professions. In the same manner, if medical students have the opportunity to interact with many people outside the medical field, they will be able to consider entrepreneurship as an option” [PE7].

*Existence of a role model*. Physician entrepreneurs argued that physicians typically work as a junior resident right after earning their medical license, and most continue to work as physicians throughout their careers. Although some might aspire to be researchers, few start a business. Thus, physician entrepreneurs pointed out that it is hard for physicians to find a role model in this area, which might prevent them from starting a business. As one physician entrepreneur mentioned, “I would like to share with the juniors the path most senior entrepreneurs have taken to make it easier for future entrepreneurs” [PE4].

#### Community

*Job security for physicians*. All respondents mentioned physicians’ job security. Physician entrepreneurs are guaranteed job security because they can concurrently work in any hospital in the country once they complete their junior residency. Therefore, they can earn a salary to make ends meet, even when their company is not yet successful enough pay their salary. One entrepreneur stated, “There is little risk in doctors’ family finances even if they try to establish a business” [PE2].

*Having spare time*. Some physician entrepreneurs insisted that having spare time can be a facilitator. Physicians with spare time can consider something different from their daily clinical work and view things from a different perspective. One participant executive officer noted, “Currently, doctors are so busy with paperwork and other administrative work that they do not have much time to focus on the practice itself, so it is crucial to develop a new system for physicians that would prevent them from being overwhelmed by their daily duties” [PE1].

#### Society

*Social trend*. Physician entrepreneurs argued that in Japan, with the aging population and the Westernization of lifestyles, lifestyle diseases were becoming critical. They also mentioned that problems with social security would be encountered in the near future, and for this reason, the industry was paying attention to medical care to determine whether anything can be done to deal with the problem. According to physician entrepreneurs, this social trend makes it easier for physicians to start a business. One entrepreneur mentioned, “Twenty years ago, it was difficult for doctors to borrow money from banks when they proposed a business plan, but now banks consider investing in the healthcare industry as a good choice. As a result, we live in an era where banks lend a lot of capital to physicians if they have a business plan” [PE5].

### Barriers

#### Individual

*Never thought about starting a business*. Most medical students seemed to think that they would become doctors and never considered starting a business, as they felt they had no talent for business and no entrepreneurship experience. During the interview, a student mentioned, “I have never thought about starting a business in my future because I am supposed to be a medical doctor. Moreover, I don’t think that I am gifted in business skills.” [MS3] Some physician entrepreneurs mentioned that they had never imagined working for a company. “Before I established my business, I believed that only those who have special talent could have started a business.” [PE2] Some physician entrepreneurs overcame this belief and started their own business; however, this belief might have narrowed a physician’s career choices.

*Misunderstanding about financial responsibility*. Nearly all medical student participants in this study believed that if they started a business and failed, they would be in debt that they could not repay. However, physician entrepreneurs insisted that the reality was that most new businesses will generally fail, and entrepreneurs can recover by taking advantage of their previous experiences. One entrepreneur mentioned, “This misunderstanding [if they failed to establish a business, they would be in debt that they could not repay] can prevent medical doctors and students from starting their own businesses” [PE2].

*Lack of business experience*. Most physician entrepreneurs and medical students mentioned that physicians and students typically lack business experience, which can hinder them from starting a new business. Physician entrepreneurs argued that most physicians and medical students in Japan had no prior experience of working for a company because most people attend medical school immediately after high school and become medical doctors directly. Thus, medical doctors tend to have limited knowledge about business. One physician entrepreneur stated that “Many physicians may have good ideas or passion but don’t know how to implement them in the form of a business” [PE2].

#### Relationship

*Objection from family members*. Physician entrepreneurs mentioned that a physician who decided to start a business had to stop working a steady job, with a consequent decrease in income. They said that they sometimes faced objections from family members. One entrepreneur insisted, “Not being able to get consent from those around you can be a problem in establishing a business. Persuading family, particularly partners, for its influence on the household’s finances can be a barrier. You can work on weekends and make a minimal income, but there’s no doubt that taking time to initiate a business will reduce your income” [PE6].

*Difficulty in recruiting suitable employees*. All the physician entrepreneurs interviewed mentioned difficulties in recruiting suitable employees. According to physician entrepreneurs, during medical school, many physicians working in Japan had little interaction with people outside of medical school and in other professions necessary to start a business. Thus, they might encounter difficulties in finding people to run their company. “People around me knew nothing about business when I decided to establish my own. To make matters worse, no one knew who I should talk to” [PE5].

*Community*. Physician entrepreneurs stated that in Japan, physician’s salaries are relatively high and stable, and this may decrease their motivation to start a business. However, most physician entrepreneurs perceived that they earned less money than when they were fully employed as physicians. According to physician entrepreneurs, from a financial viewpoint, it is hard to increase earnings until a company goes public or is acquired by another large company. One physician entrepreneur said, “It is almost impossible for physician entrepreneurs to earn more than physicians’ general salary unless they find their exit [i.e., companies go public or are acquired by a large company]” [PE7].

*Lack of diversity in physicians’ careers*. Some medical students and physician entrepreneurs perceived that almost all medical students they knew aimed to become physicians, and if medical students and physicians chose a different career, some might consider them to be dropouts. A physician entrepreneur stated, “I believed it was part of a doctor’s job to contribute to healthcare from a business side; however, physicians around me didn’t take it that way. In contrast, they considered me like a dropout. I felt sad; however, I believe that physicians will gradually change their mindset if the number of physician entrepreneurs increases” [PE5].

#### Society

*Perceived high risk of starting a business*. Medical students said they could not find a good reason to change their professions because of the high failure rate of startups in general. One student stated, “Establishing a business is as foolish as a moth flying into a flame. There’s no way any physician would choose business over working as a medical doctor” [MS4].

Both administrative officers highlighted the uniqueness of the physician workplace and healthcare-related market. As mentioned in the interviews with physician entrepreneurs and students, as there are few peers who move into working in a completely different industry since entering medical school, they rarely develop relationships with people outside the medical field; moreover, even if they develop a good idea, they often do not start a business. However, one government official pointed out that the medical market is an industry that is difficult for nonphysicians to enter without physician cooperation. As hospitals have special needs, it is the physicians who have ideas that are relevant to the field, and importantly, it is the hospitals and physicians who evaluate and introduce products that have resulted from new innovations.

All faculty members highlighted that it is not only the learning environment that is important but also the personality of the students. Two members also raised the concern that owing to the demanding medical school curriculum, spending additional time for physician entrepreneurship education would require even more time for medical students to prepare and review course materials, which may result in the loss of time and opportunities to think about their future. All faculty members also pointed out that students may not take advantage of unique career experience opportunities because they are likely to have already decided on their career paths.

## Discussion

In this study, we found that factors influencing a physician’s decision to launch a new business included their willingness to contribute to society, the unique environment in which an individual is placed while in medical school and afterward, negative aspects of the lack of diversity in physicians’ careers, financial stability provided by a medical license, and self-efficacy.

Entrepreneurs dedicated to solving problems and making society better are called social entrepreneurs [31]. All physician entrepreneurs participating in this research intended to contribute to society by solving medical problems and thus may be categorized as social entrepreneurs. Entrepreneurs generally start their business to fulfill their personal motivations [32]. Physician entrepreneurs are usually eager to cure their patients and face medical problems that need solving through their clinical work. This might explain why many of the physician entrepreneurs interviewed were motivated to improve situations they were currently facing.

We found that barriers to starting a new business included the mindset that starting a business has nothing to do with them in addition to a shortage of role models. Although there is limited research on facilitators and barriers of physician entrepreneurs [7], the importance of mindset was discussed in the context of nurse entrepreneurship [9]. This is of interest because there are similarities between physicians and nurses, such as being insiders of the healthcare system and being aware of healthcare issues, although lacking business knowledge and skills [8,9]. Most medical students we interviewed had never thought about starting a business during their career. Even physician entrepreneurs did not intend to become entrepreneurs when they were medical students. This might be partly because medical doctors and students generally have little experience in and knowledge about business, which makes it difficult for them to approach problems they face from a business perspective. Studies reported that risk, uncertainty, and lack of knowledge are major barriers to starting a new business [8,9,33], whereas another reported that lack of education, experience, moral, and financial support were subtle matters compared with mental blocks (i.e., effects of the mental block are far greater than the lack of education to starting a business in terms of preventing people from launching a new business) [34]. It would be easier to start a business if physicians had personal connections with physician entrepreneurs, although they did not have any prior business experience. However, as there are few physician entrepreneurs, medical doctors and students might hesitate to start a business. Removing these anxiety and mental barriers is crucial when providing effective support to physicians who have the intention to start a new business.

We found that the lack of diversity in the physician’s career is an important factor. This is consistent with the findings of previous studies on general entrepreneurs [35,36]. Starting a new business requires collaboration among various types of professions, and a lack of human resources often leads to failure [35,36]. However, after entering medical school, it is difficult for students to get to know people in non-physician professions, because almost all their classmates will become medical doctors. The participants perceived that this lack of diversity in the physician’s career leads to difficulties in finding people with different professions to help them.

The financial stability provided by a medical license was an important factor for physicians to launch a new business. A medical license provides rigid financial stability, because once physicians complete their junior residency, they can work in any hospital in the country. Thus, doctors often hesitate to stop their career and jump into business, and their family also sometimes objects to this challenge. As Amit and Mullar stated, dissatisfaction with positions and being lured by novel ideas are two ways to switch to self-employment [37]. The stability and lack of diversity in physician careers stated above might have contributed to keeping the number of physician entrepreneurs low, because these circumstances may never force physicians to start a business. However, the participants we interviewed perceived this stability differently. They mentioned that when people start a venture business, they will initially have no income and their finances will be tight. However, physicians can work at part-time jobs, which bring enough money to support their family budget, although their business makes no profit. This advantage of financial stability might be a unique characteristic of individuals with stable occupations, such as physicians. It would be interesting to examine whether the same tendency would be observed in people with other stable occupations.

Self-efficacy was also an important determinant, consistent with previous studies [37,38]. Mental hesitation is one of the biggest problems [34]. However, those who started their business overcame this barrier by applying their own experience [39] or drawing on the experience of other entrepreneurs. Past successes may also have a favorable impact on entrepreneurship, which is also consistent with the results of previous studies [30].

The support programs currently implemented by ministries of Japan are targeted to those who have actual ideas and are already trying to commercialize them [5,6]. These programs provide knowledge that is necessary for business, which is not taught in medical education. They also provide human resources that physicians do not encounter in their daily life and provide generous support to those who are actually moving toward entrepreneurship. However, our research shows that physicians’ current medical education and work environment may not even provide a business perspective to medical students and physicians in the first place. Thus, it may be important to remember that medical researchers might not know where to go for help or what path they need to become entrepreneurs, even if some had a good idea or research result that could be the seed of an entrepreneurial venture. Our results suggest that if we could provide medical students and physicians a sense of self-affirmation about business and a business perspective toward problem solving, it could help bring in more physician entrepreneurs. Thus, a possible support program for physician entrepreneurs might need to reduce the mental barriers and lack of perspective to begin with and provide physicians with opportunities to learn concepts and examples of entrepreneurial successes and to gain firsthand experience of the field with the help of a venture firm. A previous study that examined the impact of a technology entrepreneurship training program on subsequent entrepreneurial activities of participants (not focused on physicians) reported that participants with no entrepreneurial experience were more likely to benefit from the program than those with entrepreneurial experience [40]. As physicians often lack entrepreneurial experience, entrepreneurship training programs can be effective for physicians. Future studies examining the effects of the entrepreneurship training program for physicians, which will be developed based on the results of this study, will be needed to further facilitate entrepreneurship among physicians in Japan.

There is a limitation in the interpretation of the results of this study. First, we used the snowball sampling, and our sample primarily comprised male physician entrepreneurs in major cities and medical students in Tokyo. Female physician entrepreneurs and entrepreneurs in more rural settings may have different facilitators and barriers. However, as there are few female physician entrepreneurs in Japan, collecting data from female physician entrepreneurs until saturation is reached is challenging. Second, in this study, we interviewed physician entrepreneurs and medical students who still have potential in their careers. However, we did not conduct interviews with physicians who are not physician entrepreneurs. If we could interview physicians in future studies, we may obtain different opinions from those of medical students and thus examine in more detail the factors that promote and inhibit entrepreneurship among physicians.

## Conclusions

Our study revealed facilitators and barriers to physicians’ entrepreneurial ventures. The knowledge of these factors may be useful in supporting physicians to launch or become involved in healthcare ventures.

## Supporting information

S1 File Interview guide. Guide 1. Interview Guide for Physician Entrepreneurs. Guide 2. Interview Guide for Medical Students. Guide 3. Interview Guide for Administrative Officers at the Ministry of Health, Labour, and Welfare. Guide 4. Interview Guide for Administrative Officers at the Ministry of Economy, Trade, and Industry. Guide 5. Interview Guide for Faculty Members.
(DOCX)
