# Supplementary material for: Facilitators and barriers to physicians’ entrepreneurial ventures in major Japanese cities: A qualitative study
Source: PLoS One. 2021 Oct 27;16(10):e0258957. doi: 10.1371/journal.pone.0258957 (PMC8550390; doi:10.1371/journal.pone.0258957)
Supplement: S1 File — Guide 1. Interview Guide for Physician Entrepreneurs. Guide 2. Interview Guide for Medical Students. Guide 3. Interview Guide for Administrative Officers at the Ministry of Health, Labour, and Welfare. Guide 4. Interview Guide for Administrative Officers at the Ministry of Economy, Trade, and Industry. Guide 5. Interview Guide for Faculty Members. (DOCX) [file pone.0258957.s001.docx]

**S1 File. Interview Guide**

**Guide 1. Interview Guide for Physician Entrepreneurs**

Thank you for taking time out of your busy schedule to participate in our research. We are currently focusing on physician entrepreneurs who contribute to society through a start-up business model as a career for physicians. Through interviews with physician entrepreneurs, government officials, curriculum developers, and students, we hope to identify the knowledge and skills needed when considering entrepreneurship after becoming a physician. We also hope to identify the facilitators and barriers that influence the career path of physician entrepreneurs.

Questions

First, we would like to ask you about your experience in starting a business.

1.1 Please tell us about your career from the time you graduated until you started your own business (including your current age).

1.2 When did you start thinking about starting a business? (What was your motivation?).

1.3 Were there other people around you who started their own business?

1.4 When you were a student, did you study entrepreneurship on your own or have any internship experience?

1.5 Please tell us about your company's business (e.g., is it a system for patients or a service for medical professionals?).

We would like to ask you about undergraduate education for medical students.

2.1 If you were to provide undergraduate education to medical students, what type of course content do you think would be useful for entrepreneurship?

2.2 If you were to provide undergraduate education to medical students, how long do you think the program should be?

2.3 If you were to provide undergraduate education to medical students, students in which grade do you think it would be most suitable for?

Next, we would like to ask you about postgraduate education for doctors.

3.1 If you were to conduct a class on a postgraduate entrepreneurship support program for doctors, what do you think would be useful?

3.2 If you were to conduct a class on postgraduate support programs for doctors, how long do you think the program should be?

Finally, we would like to ask you about the actual process of starting a business.

4.1 What were the barriers to becoming an entrepreneur after becoming a doctor?

4.2 What do you think would make it easier for you to start a business after becoming a doctor, other than knowledge (e.g., skills)?

4.3 When you thought about starting a business after becoming a doctor? What type of support do you think made it easier for you to do so?

These are all the questions we have for you. Do you have any question or a suggestion? If you have, please feel free to let us know. Thank you again for your time today.

**Guide 2. Interview Guide for Medical Students**

Thank you for taking time out of your busy schedule to participate in our research. Recently, the importance of physician entrepreneurs, who contribute to society through start-up business models, has increased. Through interviews with physician entrepreneurs, government officials, curriculum developers, and students, we are considering planning and developing a (1) undergraduate education program for medical students and (2) postgraduate entrepreneurship support program for physicians to provide the knowledge and skills needed for starting a business after becoming a physician. Thus, we would like to determine the necessary knowledge and skills through interviews. To this end, we would like to interview you today to ask you, from the perspective of a student, what you think should be emphasized in entrepreneurship education.

Questions

1. If you could receive entrepreneurship education, what would you like to hear about?

2. If you could receive entrepreneurship education, in which grade would you like to take it (e.g., during liberal arts, before clinical practice, and after clinical practice)?

3. Would you like to receive entrepreneurship education?

4. Have you ever thought about starting a business?

5. What do you think are the barriers to starting a business?

6. What do you think would make it easier for you to start a business, other than knowledge (e.g., skills)?

7. What type of support do you think would make it easier for you to start a business?

If you have any question or comment, please feel free to ask us or convey. Thank you again for your time today.

**Guide 3. Interview Guide for Administrative Officers at the Ministry of Health, Labour, and Welfare**

Thank you very much for taking time out of your busy schedule to participate in our research. Recently, the importance of physician entrepreneurs, who contribute to society through start-up business models, has increased. Through interviews with physician entrepreneurs, government officials, curriculum developers, and students, we are considering planning and developing 1) a undergraduate education program for medical students and 2) an entrepreneurship support program for physicians to provide the knowledge and skills needed for starting a businesses after becoming a physician. Thus, we would like to clarify the necessary knowledge and skills through interviews. For this purpose, we would like to have an interview with you today.

Questions

1. If you were to plan a postgraduate support program for doctors from the standpoint of a university, what do you think is the most important content for supporting entrepreneurship?

2. If you were to plan a postgraduate support program for doctors from the standpoint of a university, what type of program do you think would be most appropriate (e.g., graduate school for working adults or MBA)?

3. We would like to ask you about current government programs, such as MEDISO.

3.1 What are the advantages of government programs, such as MEDISO?

3.2. What are the innovations in the management of government programs, such as MEDISO?

3.3. Was there any precedent (domestic or international) that you referred to when planning governmental programs, such as MEDISO?

4. Is there any support project that you are currently planning?

If you have any question, please feel free to ask. Thank you for your time today.

**Guide 4. Interview Guide for Administrative Officers at the Ministry of Economy, Trade, and Industry**

Thank you for taking time out of your busy schedule to participate in our research. Recently, the importance of physician entrepreneurs, who contribute to society through start-up business models, has increased. Through interviews with physician entrepreneurs, government officials, curriculum developers, and students, we are considering planning and developing 1) a undergraduate education program for medical students and 2) an entrepreneurship support program for physicians to provide the knowledge and skills needed for starting a businesses after becoming a physician. Thus, we would like to clarify the necessary knowledge and skills through interviews. For this purpose, we would like to have an interview with you today.

Questions

1. If you were to plan a postgraduate support program for doctors from the standpoint of a university, what do you think would be most important in supporting entrepreneurship?

2. If you were to plan a postgraduate support program for doctors from the standpoint of a university, what type of program (graduate school for working adults, MBA, etc.) do you think would be most appropriate?

3. Is there any program that you are currently planning?

If you have any question, please feel free to ask. Thank you for your time today.

**Guide 5. Interview Guide for Faculty Members**

Thank you for taking time out of your busy schedule to participate in our research. Recently, the importance of physician entrepreneurs, who contribute to society through start-up business models, has increased. Through interviews with physician entrepreneurs, government officials, curriculum developers, and students, we are considering planning and developing 1) a undergraduate education program for medical students and 2) an entrepreneurship support program for physicians to provide the knowledge and skills needed for starting a businesses after becoming a physician. Thus, we would like to clarify the necessary knowledge and skills through interviews. For this purpose, we would like to interview a person who is actually developing the curriculum.

Questions

1. What do you think about providing entrepreneurship education to all medical students?

2. What do you think about providing entrepreneurship education to only medical students who wish to take it?

3. First, we would like to ask you about the case where entrepreneurship education is provided to all medical students. If you were to offer entrepreneurship education to all medical students:

3.1 What type of content do you think would be feasible?

3.2 What do you think would be practically difficult?

3.3 In which grade do you think it is possible?

3.4 In which grade do you think it would be practically difficult?

3.5 How long can it be practically implemented for?

4. Next, we would like to ask you about the case of offering entrepreneurship education to only medical students who wish to take it. If you were to offer entrepreneurship education to only medical students who wish to take it:

4.1 What type of content do you think would be feasible?

4.2 What do you think would be practically difficult?

4.3 In which grade do you think it is possible?

4.4 In which grade do you think it would be practically difficult?

4.5 How long can it be practically implemented for?

If you have any question, please feel free to ask. Thank you for your time today.
